# Type D personality and metabolic syndrome among Finnish female municipal workers

**DOI:** 10.1186/s12905-020-01052-z

**Published:** 2020-09-14

**Authors:** Susa Majaluoma, Tellervo Seppälä, Hannu Kautiainen, Päivi Korhonen

**Affiliations:** 1 Institute of Clinical Medicine, Family Medicine, University of Turku and Turku University Hospital University of Turku, 20500 Turku, Finland; 2 University Hospital, Kiinamyllynkatu 4-8, 20521 Turku, Finland; 3 Health Center of Pori, Siltapuistokatu 2, 28100 Pori, Finland; 4 Department of General practice and Primary Health Care, University of Helsinki and Helsinki University Hospital, University of Helsinki, Yliopistonkatu 4, 00100 Helsinki, Finland; 5 Helsinki University Hospital, Helsingin ja Uudenmaan sairaanhoitopiiri, Stenbäckinkatu 9, PL 100, 00029 HUS Helsinki, Finland; 6 Folkhälsan Research Center, Haartmaninkatu 8, 00290 Helsinki, Finland; 7 Unit of Primary Health Care, University of Kuopio, University Hospital Kuopio. University of Kuopio, Yliopistonranta 1, 70210 Kuopio, Finland; 8 University Hospital of Kuopio, Puijonlaaksontie 2, 70210 Kuopio, Finland; 9 Central Satakunta Health Federation of Municipalities, Koulukatu 2, 29200 Harjavalta, Finland

**Keywords:** Type D personality, Metabolic syndrome, Psychosocial risk factors, Women

## Abstract

**Background:**

Type D personality is a combination of high negative affectivity (NA) and high social inhibition (SI). This personality trait is suspected to impair cardiovascular patients’ recovery. The 2016 European Guidelines on cardiovascular disease prevention in clinical practice recommend screening of psychosocial risk factors as Type D personality. The aim of this study was to assess the relationship between Type D personality and Metabolic syndrome (MetS) in working-age female population.

**Methods:**

Six hundred thirty-four female employees with mean age of 48 ± 10 years were evaluated. Type D personality and its components (NA) and (SI) were screened with DS14 questionnaire. The definition of MetS was based on measurements done by trained medical staff. We investigated the relationship between Mets and Type D personality, NA and SI using the logistic regression models adjusting for age, education years, leisure-time physical activity, smoking, alcohol use and depressive symptoms.

**Results:**

The prevalence of Type D personality was 10.6% (*n* = 67) [95% CI: 8.3 to 13.2] and MetS 34.7% (*n* = 220). Type D personality or its subcomponents were not associated with MetS. Women with Type D personality had significantly worse quality of sleep and lower LTPA. They were also more often unsatisfied with their economic situation, they had more often depressive symptoms and psychiatric disorders than non-D type persons. There were no differences in risk factors for cardiovascular diseases.

**Conclusion:**

Screening for Type D personality among working- age, reasonably healthy female population seems not to be practical method for finding persons with risk for cardiovascular disease.

## Background

Personality traits are suggested to have an impact on the etiology of Metabolic syndrome (MetS), a cluster of cardio metabolic risk factors predisposing to Type 2 diabetes and cardiovascular disease (CVD). To date the results of the studies addressing this issue are inconclusive [1].

Type D personality is combination of high negative affectivity (NA) and high social inhibition (SI). A person with Type D personality has a tendency to experience negative emotions like worry, irritability, anxiety, and to inhibit self-expression in social situations [2]. Type D personality has been found to be relatively stable over time and independent of mood or health state [2]. The prevalence of this personality type has been estimated to vary 17–38% in the general population [3, 4] and 26–53% in cardiac patients [5].

Type D personality has been suspected to impair cardiovascular patients’ recovery from cardiac events and to increase their risk of morbidity and mortality [6]. Thus the 2016 European Guidelines on CVD prevention recommend screening of psychosocial risk factors including Type D personality in clinical practice [7]. However it has not been demonstrated that Type D personality predisposes to adverse clinical outcomes in primary prevention setting. There are also gaps in the evidence for CVD prevention in women and young people because they are often underrepresented in clinical trials [7]. The aim of the present study is to assess the relationship between Type D personality and the cluster of CVD risk factors i.e. MetS, in a working-age, reasonably healthy female population. Moreover we aim to assess whether MetS is more or less common in persons with negative affectivity or social inhibition, the subcomponents of Type D personality.

## Methods

### Participants

PORTAAT (Pori To Aid against Threats) is a longitudinal study conducted among employees of the city of Pori (83,500 inhabitants in 2014) in South-Western Finland. The study population was gathered from 10 work units which were selected by the chief of the Welfare Unit of Pori. These work units had not participated in any health promotion program for a few years. The managers of the work units sent an Invitation and study information letters as an email attachment to the employees (*n* = 2570) without exclusion criteria. Information events were also organized for the employees. Altogether 836 employees consented to participate in the study. The gender distribution of the participants (104 men, 732 women) corresponded with the gender distribution of the employees of Pori at the time the study was conducted. The occupations of the participants included librarians, ground keepers, museum employees, computer works, nurses, social workers, physicians, administrative officials and general office staff. The participants were invited to baseline examinations performed by study nurses in the year 2014.

In the present analysis data from 634 female participants, who attended the PORTAAT-study in 2014 and the follow-up visit in 2015 was reported.

### Measures

Clinical measurements were performed by trained study nurses. Weight and height were measured with the participants in standing position without shoes and outer garments. Weight was measured to the nearest 0.1 kg with calibrated scales and height to the nearest 0.5 cm with a wall-mounted stadiometer. Body mass index (BMI) was calculated as weight (kg) divided by the square of height (m^2^).

Waist circumference was measured at the level midway between the lower rib margin and the iliac crest, rounded to the nearest 0.1 cm. The subjects were asked to breathe out gently at the time of measurement, and the tape was held firmly in horizontal position.

Blood pressure was measured with an automatic validated blood pressure monitor after resting at least 5 min in a sitting position. Two measurements were taken at intervals of at least 2 min and the mean of these was used in the analysis. The larger cuff was chosen if the arm circumference of the participants was > 32 cm.

Laboratory tests were analyzed from blood samples obtained after at least 8 h fasting. Plasma glucose, total cholesterol (TC), high-density lipoprotein cholesterol (HDL-C) and triglycerides (TG) were measured enzymatically (Architect c4000/c8000). Low-density lipoprotein cholesterol (LDL-C) was calculated by the Friedewald’s formula.

### Questionnaires

The participants completed self-administrated questionnaires at home before the clinical examination.

Type D personality, NA and SI were screened with questionnaire based on DS14 [1]. The DS14 consists of 14 questions; the first 7 questions define NA and the last 7 SI. The answers are chosen on a 5-point Likert scale from 0 to 4 (0 = false, 4 = true). The cut-off scale > 10 on both NA and SI scales was used to classify participants as Type D, only NA or only SI [1]. The DS14 is a valid instrument with high internal consistency and good test-retest reliability [8]. The questionnaire used in this study was the new Finnish version of the DS14 questionnaire, which was produced by the for-ward-backward translation process. Translation from English to Finnish was first performed by four native speakers of Finnish fluent in English. A native English speaker fluent in Finnish and previously unfamiliar with the DS14 translated this Finnish translation back to English. This translation was compared to the original English DS14 for conceptual equivalence.

Metabolic syndrome was defined according to the Harmonization criteria [9] as meeting at least three of the following five criteria:

1. increased waist circumference ≥ 94 cm for men, ≥80 cm for women
2. elevated blood pressure systolic ≥130 mmHg and/or diastolic ≥85 mmHg or treatment of hypertension
3. elevated triglycerides ≥1.7 mmol/l or drug treatment for elevated triglycerides
4. reduced HDL cholesterol < 1.0 mmol/l in men and < 1.3 mmol/l in women or drug treatment for reduced HDL-C
5. elevated fasting glucose > 5.6 mmol/l or diagnosed with type 2 diabetes or drug treatment of elevated glucose

Alcohol consumption was assessed with the three-item Alcohol Use Disorders Identification Test (AUDIT-C) with a cut-off value of 5 for harmful drinking [10].

Smoking status was assessed by a questionnaire. Non-smoking was defined as having never smoked or have stopped > 12 months ago.

Leisure-time physical activity (LTPA) was assessed with a questionnaire that evaluated the amount and the intensity of physical activity at leisure time. High LTPA was defined as ≥75 min vigorous intensity activities or ≥ 150 min moderate intensity activities or a combination of moderate and vigorous intensity activities per week. Moderate LTPA was defined as 1–149 min moderate or vigorous intensity activities per week and low LTPA if there were no reported moderate or vigorous intensity activities [11].

Years of education, marital status (cohabiting or not), financial satisfaction (having to spare expenditures or not) were assessed with self-administrated questionnaire. Quality of sleep (very good, good, poor or very poor) was assessed with a single question from Pittsburgh Sleep Quality index [12]. The information about regular medication and diseases diagnosed by a physician was gathered from medical records and with questionnaires. Depressive symptoms were screened with the Major Depression Inventory (MDI) [13].

### Statistical analysis

The data are presented as means with standard deviations (SD) or as counts with percentages. Characteristics of the groups were compared using the analysis of variance (ANOVA) test and chi-square test. In the case of violation of the assumptions (e.g. non-normality), a bootstrap-type test was used. The probability values for pairwise group comparisons were adjusted for multiplicity by use of the Hommel procedure. We investigated the relationship between Mets and DS14 using the logistic regression models, adjusting for age, education years, leisure-time physical activity, smoking, alcohol use, and depressive symptoms. The normality of variables was evaluated using the Shapiro–Wilk W test. Stata 15.1 (StataCorp LP; College Station, Texas, USA) statistical package was used for the analysis.

## Results

Six hundred thirty-four female employees with mean age of 48 ± 10 years were evaluated. The prevalence of Type D personality was 10.6% (*n* = 67) [95% CI: 8.3 to 13.2]. The subcomponent NA was present in 10.3% (*n* = 65) and SI in 14.7% (*n* = 93) of the participants. The characteristics of the subjects are presented in Table 1. Women with Type D personality had significantly worse quality of sleep and lower LTPA than women without Type D personality. Type D persons were also more often unsatisfied with their economic situation and they had more often depressive symptoms and psychiatric disorders than non- Type D persons. There were no differences between the participants in risk factors for cardiovascular diseases.

**Table 1:** Characteristics of the study subjects

	DS14 scale				***P***-value
	None *N* = 409	NA *N* = 65	SI *N* = 93	Type D *N* = 67	
Age, years, mean (SD)	48 (10)	49 (10)	50 (9)	49 (10)	0.52
Cohabiting, n (%)	335 (82)	51 (78)	74 (80)	49 (73)	0.39
Education years, mean (SD)	14 (2.6)	13.6 (3.0)	14.1 (2.7)	14.4 (3.0)	0.36
Weight, kg, mean (SD)	73 (14)	72 (12)	71 (14)	73 (15)	0.59
Body mass index, kg/m^2^,mean (SD)	26.9 (4.9)	26.8 (4.6)	26.6 (6.3)	26.5 (4.9)	0.91
Waist, cm, mean (SD)	89 (13)	89 (13)	88 (15)	88 (13)	0.73
Audit-C, mean (SD)	2.8 (1.6)	2.8 (1.6)	2.6 (1.5)	2.9 (1.4)	0.62
Smoking, n (%)	36 (9)	8 (12)	7 (8)	4 (6)	0.60
Good quality of sleep, n (%)	318 (78)	44 (68)	79 (85)	42 (63)	0.003*
Major Depression Inventory, mean (SD)	3.6 (4.3)	10.0 (7.6)	4.7 (3.9)	10.5 (7.6)	< 0.001*
Leisure-time physical activity, n (%)					0.018*
Low	51 (13)	18 (28)	12 (13)	13 (19)	
Moderate	192 (47)	28 (43)	55 (59)	31 (46)	
High	164 (40)	19 (29)	26 (28)	23 (34)	
Financial satisfaction, n (%)	309 (76)	42 (65)	68 (73)	40 (60)	0.024*
Regular medication, n (%)					
Hypertension	66 (16)	9 (4)	20 (22)	16 (24)	0.26
Hypercholesterolemia	18 (4)	2 (3)	6 (6)	5 (7)	0.55
Diabetes	16 (4)	1 (2)	3 (3)	2 (3)	0.92
Diseases, n (%)					
Musculoskeletal system	18 (4)	5 (8)	6 (6)	2 (3)	0.45
Cardiovascular diseases	6 (1)	1 (2)	2 (2)	1 (1)	0.95
Mental disorders	2 (1)	1 (2)	1 (1)	4 (6)	0.008
Fasting glucose, mmol/L, mean (SD)	5.49 (0.55)	5.43 (0.45)	5.52 (0.62)	5.56 (0.52)	0.46
Fasting lipids, mmol/L, mean (SD)	5.22 (0.91)	5.46 (0.92)	5.29 (0.86)	5.32 (0.99)	0.27
LDL-C	2.95 (0.74)	3.10 (0.74)	3.02 (0.73)	3.03 (0.79)	0.38
HDL-C	1.78 (0.45)	1.81 (0.45)	1.80 (0.43)	1.82 (0.43)	0.81
Triglycerides	1.11 (0.58)	1.22 (0.68)	1.05 (0.52)	1.04 (0.40)	0.23
Blood pressure, mmHg, mean (SD)					
Systolic	131 (18)	130 (17)	132 (17)	130 (16)	0.88
Diastolic	85 (11)	85 (10)	84 (10)	84 (10)	0.90

*Abbreviations*: *Audit-C* Alcohol Use Disorders Identification Test, *LDL-C* Low-density lipoprotein cholesterol, *HDL-C* High-density lipoprotein cholesterol

* Hommel’s multiple comparison procedure was used to correct significance levels for post hoc testing (*p* < 0.05):

Good quality of sleep; None/Type D, NA/SI, SI/TYPE D

Major Depression Inventory; None/NA, None/SI, None/Type D, NA/SI, SI/Type D

Leisure time physical activity; None/NA

Financial satisfaction; None/Type D

**Fig. 1:**
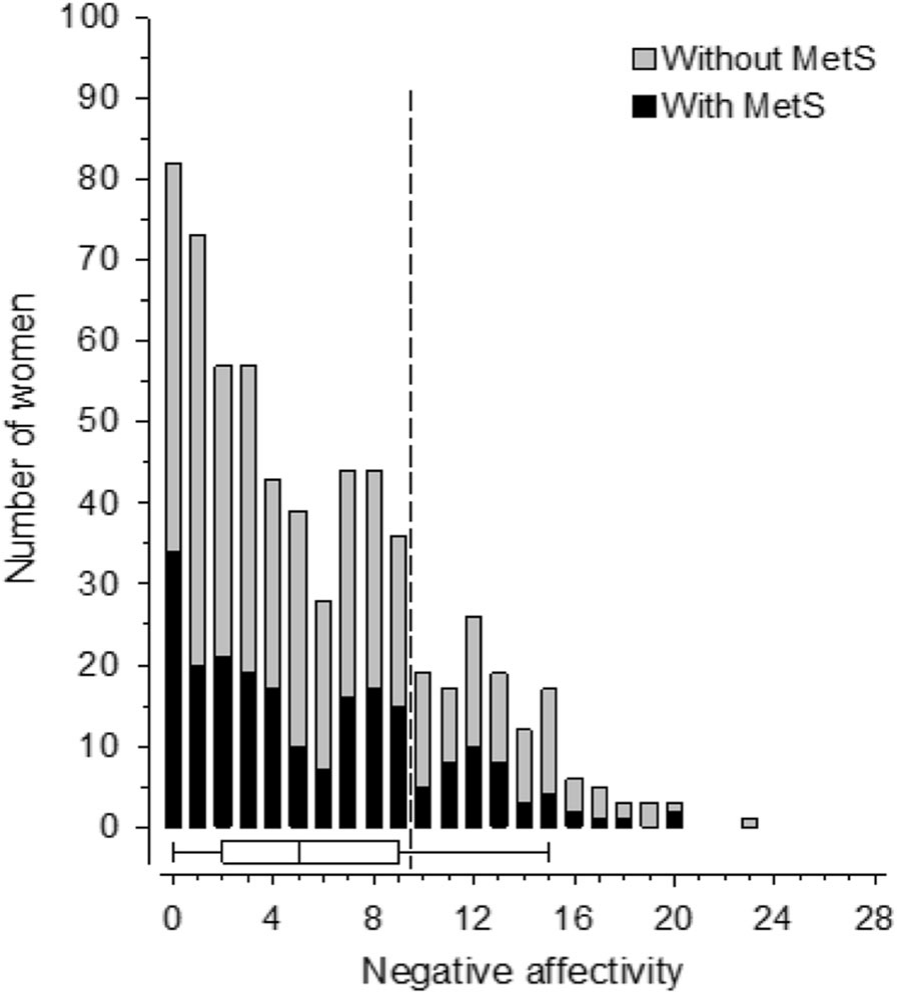
Distribution of negative affectivity scores in the study population. Box and whiskers plot shows median and interquartile range and whiskers indicate 5th and 95th percentile. Dotted line shows the cut-off score for negative affectivity

**Fig. 2:**
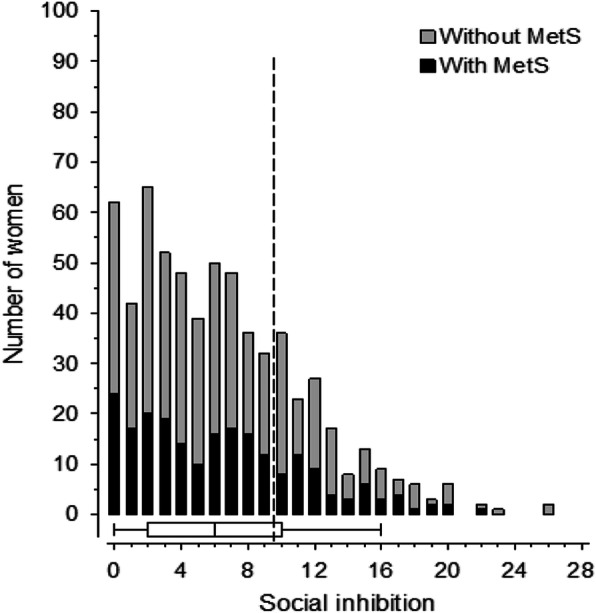
Distribution of social inhibition scores in the study population. Box and whiskers plot shows median and interquartile range and whisker indicate 5th and 95th percentiles. Dotted line shows the cut-off score for social inhibition

Figure 1 shows the distribution of NA and Fig. 2 the distribution of SI in the study population and the presence of MetS. The mean of NA score was 5, range 0–23. The mean of SI score was 6, range 0–26.

**Fig. 3:**
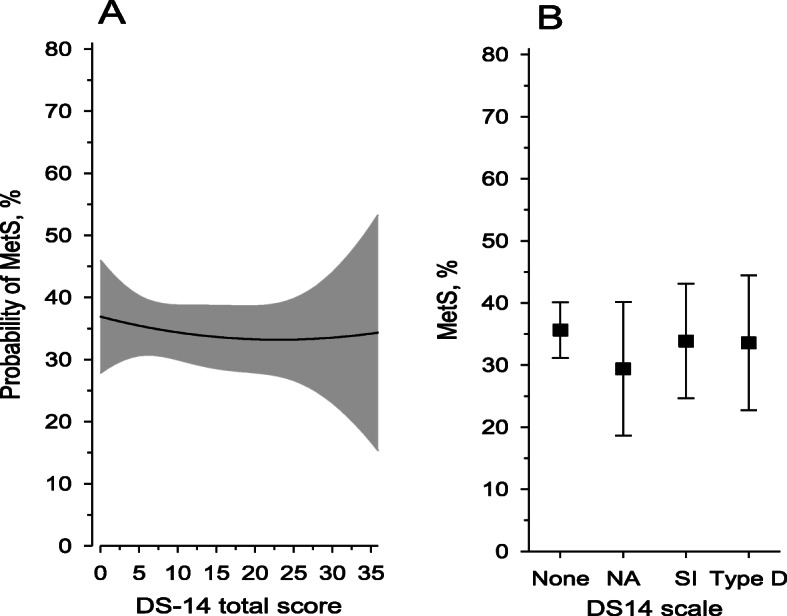
**a** Estimated probability of Metabolic syndrome according to the DS-14 total score. The curve was derived from logistic regression model. The 95% confidence intervals are denoted by gray areas. **b** Occurrence of metabolic syndrome according Type D personality and its components. Values were adjusted for age, leisure-time physical activity and education years

The prevalence of MetS was 34.7% (*n* = 220) in our study population. There were no statistical differences between women with or without Type D personality, NA or SI regarding the prevalence of MetS. Neither Type D personality nor NA or SI predicted MetS in multivariate analysis (Table 2 and Fig. 3).

**Table 2:** Type D personality and its subcomponents as predictors of Metabolic syndrome

	Model 1^a^		Model 2^b^		Model 3^c^	
	OR (95% CI)	*P*-value	OR (95% CI)	*P*-value	OR (95% CI)	*P*-value
DS14 scale						
None	1 (reference)		1 (reference)		1 (reference)	
Negative affectivity	0.82 (0.45–1.48)	0.51	0.77 (0.42–1.42)	0.40	0.65 (0.34–1.26)	0.20
Social inhibition	0.92 (0.56–1.51)	0.73	0.89 (0.53–1.47)	0.64	0.83 (0.50–1.38)	0.46
Type D personality	0.94 (0.53–1.67)	0.83	0.90 (0.50–1.61)	0.73	0.77 (0.41–1.45)	0.41

^a^Model 1 adjusted for age and education years

^b^Model 2 adjusted for age, education years, leisure-time physical activity, smoking and alcohol use

^c^Model 3 adjusted for age, education years, leisure-time physical activity, smoking, alcohol use and depressive symptoms

## Discussion

The present study indicates that although Type D personality and MetS are quite common in working- age women, there is no association with Type D personality or its subcomponents and MetS. Instead participants with Type D personality slept worse, reported LTPA, had more depressive symptoms and were more dissatisfied with their financial situation than subjects without Type D personality. The negative way of thinking and cynicism might at least partly explain these differences. In our study population, one out of 10 women had Type D personality and about a third of the participants had MetS. The prevalence of Type D is relatively low in the study population, but to our knowledge, this is the first study assessing Type D personality in Finland.

There are few studies concerning Mets and Type D personality.

In the Netherlands, Mommersteeg et al. detected a twofold increased risk of having Mets in persons with Type D personality [14]. The study population was 1592 participants from general population aged 20–80 years. Mets was defined by self-report and the prevalence of MetS was 7.3%.

In Greece, Tziallas et al. also found an association between Type D personality and MetS [15]. The study population consisted of 359 patients of an outpatient lipid clinic in a University Hospital. In this highly selected population the prevalence of Mets was 57.4%.

Findings in the present study are similar with a German study among 458 workers (80% men) of an airplane manufacturing company [16]. The prevalence of Type D personality was 32% (30% in men, 38% in women) and the prevalence of Mets 14%. Type D personality, NA or SI were related to Mets neither in cross-sectional nor longitudinal (mean follow-up time 6.3 years) research frame [16].

The major limitation of our study is its cross-sectional nature and thus any causal relationship between Type D personality and MetS cannot be done. Although no association between Type D personality and Mets was found, the situation might be different in 5–10 years. Self-reporting of diet, physical activity and smoking status may also be unreliable, but we use validated questionnaires and standardized procedures in order to overcome this bias. We excluded male employees because there were so few of them and thus the results and interpretations cannot be generalized to men. However the mean age and gender distribution among the PORTAAT study participants resemble the distribution of all employees of the city of Pori and also Finnish public sector employees overall [17]. The mean annual rate of sickness absence days among the study participants was comparable to the non-participants in the included employment sectors [18]. The response rate of 32.5% is relatively low, but it is known that E-mail surveys generally have about 20% lower response rate than mail surveys [19]. It is also possible that person with Type D personality are reluctant to attend this kind of survey indicating lifestyle related factors. People with better health are more likely to attend health surveys and thus the healthy worker effect is possible in our study. The prevalence of psychiatric disorders is low (1.3%) in this study population of active work force. Even though previously detected diseases were assessed both through register data and by clinical interview, there is a possibility that psychiatric disorders were underdiagnosed. The strength of our study is that the clinical measurements were done by trained medical staff and consensus definition of MetS was used. Also many aspects of health related behaviors was taken into account.

## Conclusion

Screening for Type D personality in working-age, relatively healthy female population seems not to be practical method for finding persons at risk for CVD or Type 2 diabetes. Although we found no association between Type D personality and MetS, the subcomponent negative affectivity may be related to depressive symptoms and sleep disorders among female employees. This finding warrants for further investigations about the impact of Type D personality on individuals’ mental health and quality of life.

## Data Availability

The datasets during and/or analyzed during the current study available from the corresponding author on reasonable request.

## Abbreviations

CVD: Cardiovascular disease
NA: Negative affectivity
SI: High social inhibition
MetS: Metabolic syndrome
LTPA: Leisure time physical activity
BMI: Body mass index
TC: Total cholesterol
HDL-C: High-density lipoprotein cholesterol
TG: Triglycerides
LDL-C: Low-density lipoprotein cholesterol
AUDIT-C: Alcohol Use Disorders Identification Test
MDI: The Major Depression Inventory
